# P-613. Direct Impact of Pediatric Immunization on Reduction in Antibiotic Prescribing in the United States

**DOI:** 10.1093/ofid/ofae631.811

**Published:** 2025-01-29

**Authors:** Justin Carrico, Sandra E Talbird, Amanda Eiden, Cristina Carias, Min Huang, John C Lang, Gary S Marshall, Goran Bencina

**Affiliations:** RTI Health Solutions, Research Triangle Park, North Carolina; RTI Health Solutions, Research Triangle Park, North Carolina; Merck, Philadelphia, PA; Merck & Co., Inc., Rahway, New Jersey; Merck & Co., Inc., Rahway, New Jersey; Merck Canada Inc., Kirkland, QC, Canada, Kirkland, Quebec, Canada; Norton Children's and University of Louisville School of Medicine, Louisville, KY; MSD, Madrid, Madrid, Spain

## Abstract

**Background:**

Immunization programs, by preventing infectious diseases for which antibiotics are prescribed, have the potential to reduce antibiotic use and development of antimicrobial resistance. However, limited data are available regarding the impact of vaccines on antibiotic prescribing at the population level. This study evaluated the potential impact of pediatric immunization on prescribed antibiotic treatment courses in the United States (US).

**Table 1.**
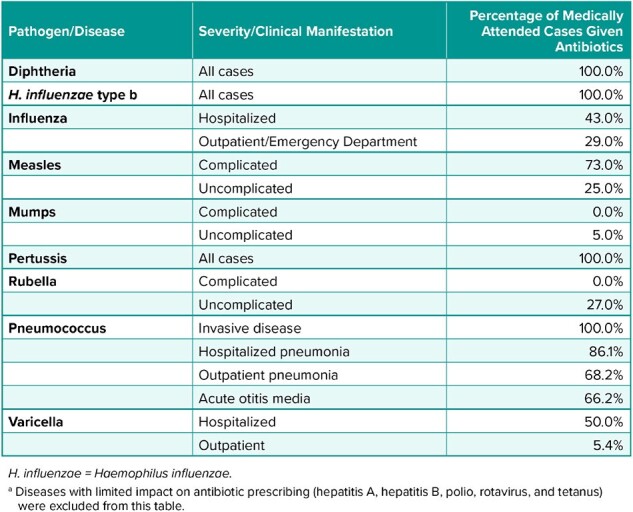
Antibiotic Usage Assumptions by Disease

**Methods:**

A previously published decision tree model was used to estimate disease cases averted in the total US population by the routine childhood immunization schedule. Antibiotic courses for the 14 vaccine-preventable diseases (VPDs) covered by the US immunization schedule estimated from disease- or syndrome-specific real-world prescribing rates (where available) and clinical practice guidelines (Table 1); importantly, real-world prescribing rates include antibiotics prescribed for viral VPD cases, whether or not secondary bacterial infections are definitively diagnosed. Antibiotic prescription rates were multiplied by disease cases averted to estimate antimicrobial courses averted in a single year for the 2023 total US population.

**Table 2.**
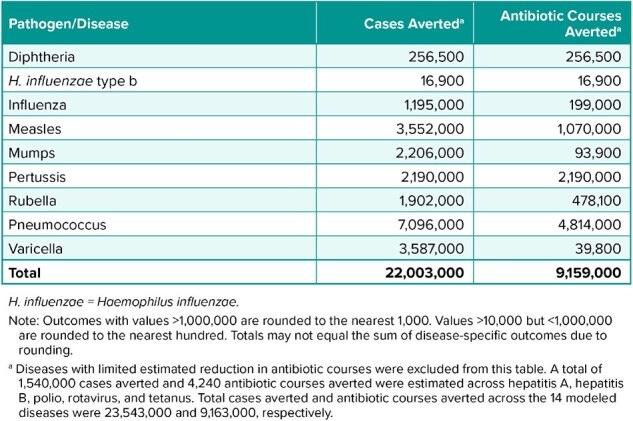
Annual Reduction in Antibiotic Treatment Courses for the 2023 US Population

**Results:**

Routine childhood immunization was estimated to have averted 9.2 million annual antibiotic courses in the 2023 US population (Table 2). The number of antibiotic courses averted were largest for pneumococcus (4.8 million), pertussis (2.2 million), and measles (1.1 million). Over 90% reduction in antibiotic courses was estimated for 8 of the 14 modeled VPDs. Residual annual antibiotic courses due to VPDs in 2023 remained highest for pneumococcus (1.6 million), influenza (0.9 million), and pertussis (0.2 million).

**Conclusion:**

Routine pediatric immunization in the US results in reduced annual antibiotic prescribing. The estimates provided herein represent only the direct impact on antibiotic prescribing because the model did not account for potential reductions in antibiotic prescribing for fully vaccinated children presenting with nonspecific febrile illnesses, in whom the likelihood of bacterial VPD would be lower.

**Disclosures:**

**Justin Carrico, BS**, Merck & Co., Inc.: Advisor/Consultant **Sandra E. Talbird, MSPH**, Merck & Co., Inc.: Advisor/Consultant **Amanda Eiden, PhD, MBA, MPH**, Merck & Co., Inc.: Stocks/Bonds (Private Company) **Cristina Carias, PhD**, Merck & Co., Inc.: Stocks/Bonds (Private Company) **Min Huang, PhD**, Merck & Co., Inc.: Employee|Merck & Co., Inc.: Stocks/Bonds (Public Company) **John C. Lang, PhD, MSc, MSc, BSc**, Merck & Co., Inc.: Stocks/Bonds (Private Company)|Merck Canada Inc.: Employee **Gary S. Marshall, MD**, GSK: Advisor/Consultant|GSK: Grant/Research Support|GSK: Honoraria|Merck & Co., Inc.: Advisor/Consultant|Merck & Co., Inc.: Grant/Research Support|Merck & Co., Inc.: Honoraria|Moderna: Advisor/Consultant|Moderna: Honoraria|Pfizer: Advisor/Consultant|Pfizer: Grant/Research Support|Pfizer: Honoraria|Sanofi: Advisor/Consultant|Sanofi: Grant/Research Support|Sanofi: Honoraria|Seqirus: Advisor/Consultant|Seqirus: Grant/Research Support|Seqirus: Honoraria **Goran Bencina, PhD**, Merck & Co., Inc.: Stocks/Bonds (Private Company)

